# The Outcome of Electrical Cardioversion in Hyperthyroid Induced Atrial Fibrillation

**DOI:** 10.7759/cureus.37928

**Published:** 2023-04-21

**Authors:** Hriday Shah, Kerollos S Hanna, Harkirat Kaur, Mohammad S Alazzeh, Abhay Thandavaram, Aneeta Channar, Ansh Purohit, Bijay Shrestha, Deepkumar Patel, Lubna Mohammed

**Affiliations:** 1 Internal Medicine, California Institute of Behavioral Neurosciences & Psychology, Fairfield, USA; 2 General Physician, California Institute of Behavioral Neurosciences & Psychology, Fairfield, USA; 3 Orthopaedic Surgery, California Institute of Behavioral Neurosciences & Psychology, Fairfield, USA; 4 Family Medicine, California Institute of Behavioral Neurosciences & Psychology, Fairfield, USA; 5 Neurology, California Institute of Behavioral Neurosciences & Psychology, Fairfield, USA

**Keywords:** thromboembolic events, recurrence rate, hyperthyroidism, atrial fibrillation, electrical cardioversion

## Abstract

Hyperthyroidism is a prevalent cause of atrial fibrillation (AF). High cardiac output with low systemic vascular resistance, driven by hyperthyroidism, is associated with a rapid heartbeat, enhanced left ventricular systolic and diastolic function, and a higher incidence of supraventricular tachyarrhythmias. After returning to euthyroid status, hyperthyroidism-induced AF generally spontaneously reverts to sinus rhythm (SR), and a significant number of patients remain in chronic AF and require electrical cardioversion (ECV). After effective cardioversion, the long-term outcome of hyperthyroidism-induced persistent AF is unknown. Early ECV before the antithyroid medication should be explored for hyperthyroidism-induced AF to reduce the risk of thromboembolic consequences. The recurrence rate of AF between Hyperthyroid and Euthyroid Patients after ECV did not significantly differ. This review article compares the recurrence rate of AF as an outcome of ECV in Hyperthyroid induced atrial fibrillation.

## Introduction and background

Acute atrial fibrillation (AF) is fast, irregular, and inchoate atrial activity. Increasing age, cardiovascular illness, alcohol, diabetes, and lung disease are all risk factors for acute AF. Acute AF raises the risk of cardiac failure and stroke. In more than half of the cases, the illness resolves within 24 to 48 hours; however, many others will require medical intervention to regulate heart rate or restore sinus rhythm (SR) [1]. The world's most common arrhythmia, AF, is increasingly prevalent as people age [2]. Hyperthyroidism is a well-known cause of atrial fibrillation, with an incidence of 16% to 60% in patients with confirmed hyperthyroidism [2].

Frost et al. looked for a new-onset diagnosis of AF or Atrial flutter (AFL) in 40 628 people with new-onset hyperthyroidism who were being observed in an inpatient setting for 30 days following their hyperthyroidism diagnosis [3]. 8.3% of these patients reported an AF or AFL diagnosis with a new onset [3]. Thyroid hormones may raise the left atrial pressure due to increased left ventricular mass (LVM) and decreased ventricular relaxation, cause ischemia through increased resting heart rate, and increase ectopic atrial activity, among other potential mechanisms.

Research employing an isolated heart model has demonstrated that experimental thyrotoxicosis animals' hearts beat rapidly and had shorter mean adequate refractory periods than euthyroid animals' hearts [4]. The hyperdynamic cardiovascular condition that hyperthyroidism causes (high cardiac output with low systemic vascular resistance) is linked to a rapid heart rate, improved left ventricular systolic and diastolic function, and a higher prevalence of supraventricular tachyarrhythmias [4]. The heart rate was higher, and the turbulence slope (TS), which measures the rate at which the sinus slows after sinus tachycardia, was lower in hyperthyroid patients, indicating reduced vagal tone [4].

The first external electrical cardioversion (ECV) procedure occurred in the 1950s. This early experience showed that electrical energy sent to the thorax from the outside could activate the heart. Zoll et al. effectively terminated ventricular fibrillation in a patient using externally administered defibrillation shocks [5]. According to Kouwenhoven and colleagues [6], a battery-powered direct current-driven device might be portable. Electrical counter-shocks can be delivered using either alternating current (AC) or direct current (DC) energy [7]. ECV can treat AF and AFL to achieve SR [7]. AF caused by hyperthyroidism typically spontaneously returns to SR after the patient is in a euthyroid condition, but many people continue to go through chronic AF and need ECV. After efficacious cardioversion, the long-term outcome of hyperthyroidism-induced persistent AF is unknown [8]. This review aims to assess ECV's effectiveness in hyperthyroidism-induced AF.

## Review

Hyperthyroidism and atrial fibrillation

Thyrotoxicosis is the most prevalent treatable cause of AF, with a confirmed 5-fold increased risk. In hyperthyroidism and triiodothyronine (T3) toxicosis, the elderly are more likely to have AF. Low Thyroid Stimulating Hormone (TSH) levels correlate positively with the risk of AF, and subclinical hyperthyroidism is associated with a threefold increase in AF [9]. A low blood thyrotropin concentration is linked to a three-fold increased chance of developing AF within a decade in adults 60 years or older [9]. In a study done involving over 23000 people, AF was found in 513 participants (2.3 percent) in the group with normal blood TSH values, 78 (12.7 percent) in the group with subclinical hyperthyroidism, and 100 (13.8 percent) in the group with overt hyperthyroidism [4]. Serum-free thyroxine (fT4) was discovered to be an independent predictor of the prevalence of AF in the total population in a study by Gammage et al. [10]. This connection persisted even after people with overt thyroid disease were eliminated.

Furthermore, the association between serum fT4 and AF remained obvious when the analysis was restricted to individuals categorized as euthyroid when blood TSH levels were normal [4,10]. Patients with new-onset AF were followed in the outpatient setting for 13 years in a large nationwide cohort study conducted in Denmark by Selmer and colleagues to see if they developed hyperthyroidism [11]. 

Compared to the general population of that age without a diagnosis of AF, a considerably greater incidence of hyperthyroidism was detected throughout the 13-year follow-up, particularly in the between-the-ages-of-51 and 60 male groups [11]. AF can lead to worsening cardiac health, particularly in the context of pre-existing heart disease, and it is also linked to embolic consequences, particularly cerebral embolism. These factors will likely affect the higher cardiovascular and cerebrovascular mortality mentioned earlier. Furthermore, in people with AF complicating thyrotoxicosis, the likelihood of spontaneous restoration of SR decreases with age, most likely reflecting the prevalence of underlying ischemic, hypertensive, or valvular heart disease [12].

Pathophysiology

The heart's β1-adrenergic and M2-muscarinic receptors are altered by high thyroid hormone in hyperthyroid patients, resulting in enhanced sympathetic activity, tachycardia, and a shorter atrial refractory time. The thyroid hormone is also known to function in ionic channel alteration. The effects of thyroid hormone on messenger ribonucleic acid (mRNA) expression and currents of key ionic channels in murine atria were investigated by Watanabe et al. [13]. The authors discovered that thyroid hormone caused such drastic alterations [13].

Hyperthyroidism increases the resting heart rate, blood volume, stroke volume, myocardial contractility, and ejection fraction, which is analogous to an increase in adrenergic activity. Additionally, diastolic relaxation is improved in this condition. Furthermore, the therapeutic benefits of beta-blockers show that elevated catecholamine activity is the root cause of the cardiac symptoms of hyperthyroidism. Plasma catecholamine levels are low or unaltered in thyrotoxicosis, whereas tissue-dependent and time-dependent increases in adrenergic receptor density improve the tissue's sensitivity to catecholamines. To maintain cellular sensitivity to beta-1 adrenergic agonists, thyroid hormone decreases the expression of cardiac-specific adenylyl cyclase catalytic subunit isoforms and increases the number of beta-1 adrenergic receptors and guanosine triphosphate binding proteins [14].

The development of AF in hyperthyroidism may be affected by several factors, including elevated left atrial pressure, which increases left ventricular mass and inhibits ventricular relaxation, elevated resting heart rate-induced ischemia, and elevated atrial ectopic activity. Premature complexes in the pulmonary veins are the origin of AF, and a re-entry pathway is required for their persistence [4,14]. Instead of paroxysmal, this kind of AF is typically persistent [15].

Effect of persistent atrial fibrillation

AF is an independent risk factor for thromboembolic events [16], and it has a 5-7 times greater chance of thromboembolic outcomes (3-6%) than those with SR. There is an increased risk of blood clots inside the heart with AF, which can lead to embolism, stroke, silent cerebral infarction, and other problems because of the rapid and irregular heartbeat [16,17]. According to Siu et al., those with hyperthyroidism who develop new-onset AF are more likely to have an ischemic stroke immediately after diagnosis. Consequently, hyperthyroidism-induced AF should be treated immediately [17,18]. 

Electrical cardioversion

ECV is usually done with continuous Electrocardiogram (ECG) monitoring and intravenous procedural anesthetic; resuscitation procedures should be considered due to the danger of ventricular fibrillation and asystole. The defibrillator/cardioverter electrodes can be positioned anterior-left lateral (apical) or anterior-posterior such that the heart is between the two electrodes. With early monophasic defibrillators, the anterior-posterior arrangement was the most effective approach to position the electrodes [19]. Thanks to biphasic defibrillators, higher cardioversion rates can now be obtained at lower energy levels [20].

1.7% of newly hyperthyroid people had persistent AF [21]. The initial strategy is to begin antithyroid therapy as soon as possible and regularly administer pharmacologic therapy to control heart rate. Because more than 56% of AF spontaneously converts to SR when thyroid hormone levels start to fall, cardioversion should be postponed until the fourth month of maintaining a euthyroid state. With SR maintenance rates of 56.7% and 47.6% in the 10th and 14th years, respectively, elective cardioversion is beneficial for those whose AF persists [21], even though the length of AF previous to cardioversion was unusually lengthy (35.0 +/- 29.0 months).

Electrical cardioversion in hyperthyroid-induced atrial fibrillation

Since the length and severity of atrial stunning are significantly influenced by the duration and intensity of AF, restoring SR as soon as it is practically possible reverses atrial stunning and reduces the risk of thromboembolic consequences. To reduce the risk of thromboembolic consequences, early ECV before the antithyroid medication should be explored for hyperthyroidism-induced AF [17].

Nakazawa et al. [22] assessed the outcome of cardioversion in patients with thyrotoxicosis-induced AF throughout a median 28.5-month period. At a lengthy follow-up (mean 80.6 months), they reported that, of the 106 patients, 98 had been successfully cardioverted, and 67% were still in SR [22]. According to a study by Siu et al. [18], after a successful ECV, the AF recurrence rate for patients with hyperthyroidism-induced AF was 59% as opposed to 83% for non-thyroid AF (p = 0.04) over a 24-month follow-up period [8]. One month after cardioversion, 62.9% of euthyroid people and 63.3% of hyperthyroid patients in a study by Ari et al. [17] were at SR. In their research, the one-month short-term recurrence rate of AF following cardioversion was comparable to those documented in the literature [17,23].

According to Ari et al. [23], 20 of 58 patients (34.5%) who had chronic AF reverted to AF six months following a successful ECV. In 78 patients with persistent AF, Vikman et al. [24] found a 35% one-month recurrence rate following the restoration of SR with ECV. During a month of follow-up, Tieleman et al. [25] found a higher rate of recurrence: 35 out of 61 patients (57%) in their research experienced an AF relapse. Another fascinating finding of a study by Ari et al. [17] is that the AF recurrence rate was substantially greater in clinically apparent hyperthyroidism than in subclinical hyperthyroidism at one month (52.6 and 9.1%, respectively, p = 0.023). Regarding AF recurrence rate, neither the clinically evident hyperthyroidism group nor the subclinical hyperthyroidism group (p = 0.27 and p = 0.13, respectively) showed statistically significant differences from the euthyroid control group. According to this study, early ECV can prevent AF recurrence in people with both clinical and subclinical hyperthyroidism, albeit it might be more effective in those with subclinical hyperthyroidism.

**Figure 1:**
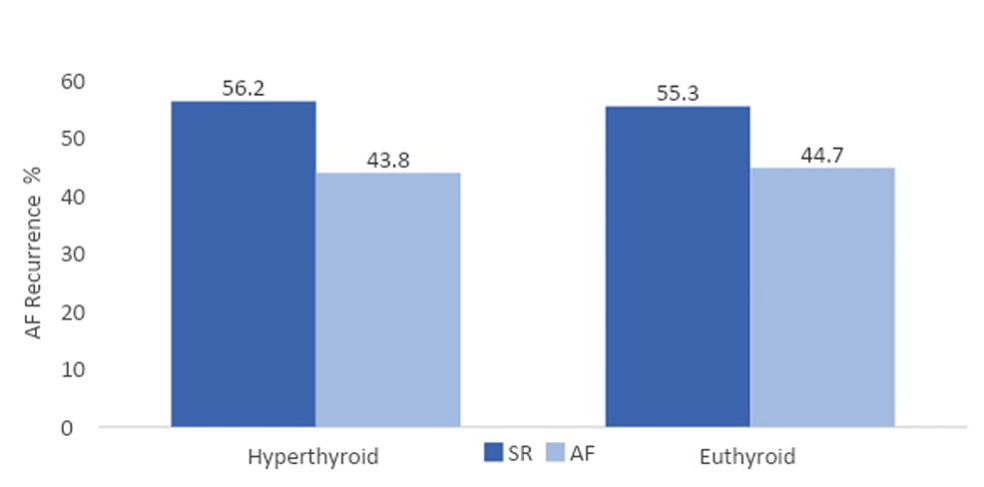
Rates of atrial fibrillation recurrence in hyperthyroid and euthyroid patients. p=0.93 for Hyperthyroid and Euthyroid groups SR: sinus rhythm; AF: atrial fibrillation Adapted from Gurdogan et al. [26]

In a study by Gurdogan et al., the average follow-up for the hyperthyroid group was 23.63 ± 3.74 months, while the average follow-up for the euthyroid group was 22.78 ± 3.15 months (p = 0.51); 14 (43.8%) and 21 (44.7%) individuals in each group relapsed with AF (p = 0.93) represented in (Figure 1) [26]. With odds ratios of 1.38 (95% Confidence Interval [CI] = 1.05 - 1.82, p = 0.02) in the hyperthyroid group and 1.42 (95% Confidence Interval [CI] = 1.05 - 1.91, p = 0.02) in the euthyroid group, the length of AF was the sole factor that predicted AF recurrence in both groups [26]. The studies that compare the frequency of recurrences and follow-up intervals in persistent AF are shown in (Table 1) for comparison.

**Table 1:** Compares rate of recurrences and follow-up periods after cardioversion in persistent atrial fibrillation by various studies. Created by the author.

Study	Follow-up Period	Recurrence Rate
Nakazawa et al. [22]	80.6 months	NR
Siu et al. [8]	24-months (Hyperthyroid group)	59%
Ari et al. [17]	6-months	34.5%
Gurdogan et al. [26]	23.63-months (hyperthyroid group)	43.8%
Vikman et al. [24]	1 month	35%
Tieleman et al. [25]	1month	57%

Due to the risk of atrial stunning, cardioversion may be an option for those who remain in AF after 8-10 weeks of remaining in a euthyroid state with anticoagulation for at least three weeks. It is crucial to highlight that the odds of unsuccessful cardioversion drastically rise with time in individuals with AF who are euthyroid or hyperthyroid at the time of diagnosis. One year after diagnosis, there is a high probability of recurrence, and the risk is significantly higher two years later. A longer duration of AF before cardioversion was a poor predictor of maintaining SR in a study conducted by Gurdogan and colleagues, regardless of thyroid status [2].

Complications associated with electrical cardioversion

**Figure 2:**
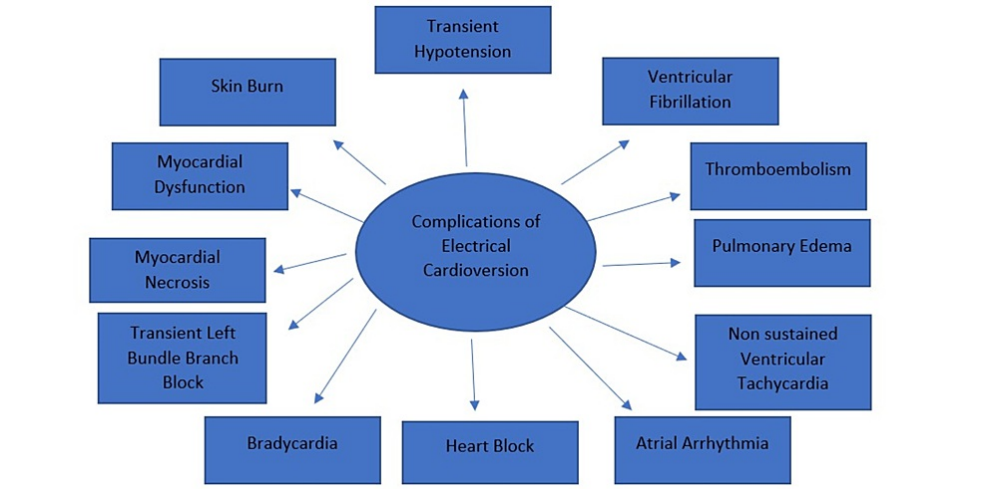
Complications of Electrical Cardioversion. Created by author.

Complications of ECV for AF are uncommon. Possible side effects include thromboembolism from inadequate anticoagulant therapy, general anesthesia-induced ventricular fibrillation (VF), non-sustained ventricular tachycardia (VT), atrial arrhythmia, heart block, bradycardia, transient left bundle branch block, myocardial necrosis, myocardial dysfunction, transient hypotension, pulmonary edema, skin burn, and transient left bundle branch block. Pain at the application location is correlated with the number of applications. In stable patients with a left atrial thrombus and insufficient anesthetic, the ECV is contraindicated [7]. Figure 2 shows the complications associated with Electrical Cardioversion.

Thromboembolic events associated with electrical cardioversion

Cardioversion for AF or AFL is usually safe but is associated with a higher risk of thromboembolic incidents. The risk of thromboembolic events does not appear to differ between Pharmacological Cardioversion (PCV) and Electrocardiographic Cardioversion (ECV) or AF and AFL. Thromboembolic event after cardioversion is defined as the creation and subsequent embolization of new thrombi in the atrium that arise when the atrial function is still compromised in the weeks following cardioversion or the embolization of pre-existing thrombi in the atrium at the time of cardioversion [27].

Temporal relationship of thromboembolic events after cardioversion

Data from 32 trials (published up to 1997) and 4621 patients, including 92 patients who had a thromboembolic event after ECV, were used by Berger and Schweitzer [28] to analyze the timing of thromboembolic events that followed ECV of AF or AFL. There were around one to eighteen days between cardioversion and thromboembolic occurrences. Seventy-five (82%) of the 92 embolic occurrences happened three days later, 88 (96%) within a week, and 90 (98%) within ten days after the ECV. Therefore, current recommendations recommend anticoagulation for up to four weeks after cardioversion [29]. Peri-procedural thromboembolism can be caused by several conditions, including pre-existing thrombi, transient atrial stunning after cardioversion, alterations in mechanical atrial systolic function, enlarged left atrium, and prothrombotic state [27].

Limitations

Cardioversion in persistent AF due to hyperthyroidism needs further randomized studies. Studies included in this review article had a small sample size. Furthermore, studies with longer follow-up periods are required to know the AF recurrence rates.

## Conclusions

Hyperthyroidism is the most frequent, curable, and preventable cause of AF. The development of AF in hyperthyroidism may be influenced by various causes, including elevated left atrial pressure, which increases left ventricular mass and impairs ventricular relaxation, ischemia brought on by a rapid resting heart rate, and elevated atrial ectopic activity. Atrial fibrillation is one of the Independent risk factors for thromboembolic events. Particularly in subclinical hyperthyroidism individuals without waiting for a euthyroid state, early electrical cardioversion should be considered for hyperthyroidism-induced AF. The risk of thromboembolic effects would be decreased by quickly restoring SR. Although cardioversion for AF or AFL is generally considered safe, there has been evidence linking the procedure to an increased risk of thromboembolic events. As a result, current recommendations suggest using anticoagulants for up to four weeks following cardioversion. More prospective trials with a large sample size are required to prove the advantages of early electrical cardioversion during the treatment strategy for AF caused by hyperthyroidism.
